# Alcohol, tobacco and breast cancer – collaborative reanalysis of individual data from 53 epidemiological studies, including 58 515 women with breast cancer and 95 067 women without the disease

**DOI:** 10.1038/sj.bjc.6600596

**Published:** 2002-11-12

**Authors:** Collaborative Group on Hormonal Factors in Breast Cancer

**Affiliations:** Secretariat, Cancer Research UK Epidemiology Unit, Gibson Building, Radcliffe Infirmary, Woodstock Road, Oxford OX2 6HE, UK

**Keywords:** breast cancer, alcohol, tobacco, smoking, collaborative reanalysis

## Abstract

Alcohol and tobacco consumption are closely correlated and published results on their association with breast cancer have not always allowed adequately for confounding between these exposures. Over 80% of the relevant information worldwide on alcohol and tobacco consumption and breast cancer were collated, checked and analysed centrally. Analyses included 58 515 women with invasive breast cancer and 95 067 controls from 53 studies. Relative risks of breast cancer were estimated, after stratifying by study, age, parity and, where appropriate, women's age when their first child was born and consumption of alcohol and tobacco. The average consumption of alcohol reported by controls from developed countries was 6.0 g per day, i.e. about half a unit/drink of alcohol per day, and was greater in ever-smokers than never-smokers, (8.4 g per day and 5.0 g per day, respectively). Compared with women who reported drinking no alcohol, the relative risk of breast cancer was 1.32 (1.19–1.45, *P*<0.00001) for an intake of 35–44 g per day alcohol, and 1.46 (1.33–1.61, *P*<0.00001) for ⩾45 g per day alcohol. The relative risk of breast cancer increased by 7.1% (95% CI 5.5–8.7%; *P*<0.00001) for each additional 10 g per day intake of alcohol, i.e. for each extra unit or drink of alcohol consumed on a daily basis. This increase was the same in ever-smokers and never-smokers (7.1% per 10 g per day, *P*<0.00001, in each group). By contrast, the relationship between smoking and breast cancer was substantially confounded by the effect of alcohol. When analyses were restricted to 22 255 women with breast cancer and 40 832 controls who reported drinking no alcohol, smoking was not associated with breast cancer (compared to never-smokers, relative risk for ever-smokers=1.03, 95% CI 0.98–1.07, and for current smokers=0.99, 0.92–1.05). The results for alcohol and for tobacco did not vary substantially across studies, study designs, or according to 15 personal characteristics of the women; nor were the findings materially confounded by any of these factors. If the observed relationship for alcohol is causal, these results suggest that about 4% of the breast cancers in developed countries are attributable to alcohol. In developing countries, where alcohol consumption among controls averaged only 0.4 g per day, alcohol would have a negligible effect on the incidence of breast cancer. In conclusion, smoking has little or no independent effect on the risk of developing breast cancer; the effect of alcohol on breast cancer needs to be interpreted in the context of its beneficial effects, in moderation, on cardiovascular disease and its harmful effects on cirrhosis and cancers of the mouth, larynx, oesophagus and liver.

*British Journal of Cancer* (2002) **87**, 1234–1245. doi:10.1038/sj.bjc.6600596 www.bjcancer.com

© 2002 Cancer Research UK

## 

Many epidemiological studies have investigated the relationship between breast cancer and the consumption of alcohol and/or tobacco. References to over 80 studies that have collected relevant data, as well as to reviews of the subject, are given in Appendix II (www. bjcancer.com). The published results from these studies have generally suggested that women who regularly consume alcohol may be at a slightly increased risk of the disease, but the findings reported for tobacco are inconsistent. Alcohol and tobacco consumption are known to be associated one with another, and published results have not always allowed adequately for possible confounding between these exposures. Individual data from 65 epidemiological studies of breast cancer 63 published^1,2,3,4,5,6,7,8,9,10,11,12,13,14,15,16,17,18,19,20,21,22,23,24,25,26,27,28,29,30,31,32,33,34,35,36,37,38,39,40,41,42,43,44,45,46,47,48,49,50,51,52,53,54,55,56,57,58,59,60,61,62,63^ and two unpublished in which information on alcohol and/or tobacco consumption had been collected contributed to this collaboration. These studies, some of which have not published results for alcohol or tobacco, include over 80% of the worldwide information on the topic (see Appendix II (www.bjcancer.com)). The data from these studies were analysed, taking careful account of the possible confounding between alcohol and tobacco consumption, as well as confounding by other factors.

## METHODS

### Eligibility of studies and collection of data

Data from epidemiological studies of women with breast cancer have been brought together by the Collaborative Group on Hormonal Factors in Breast Cancer to describe the relationship between breast cancer and various reproductive, hormonal and other factors.^64,65^ Case–control and cohort studies were eligible for the collaboration if they included at least 100 women with incident invasive breast cancer and recorded information on reproductive factors and on use of hormonal therapies. Cohort studies were included using a nested case–control design, in which four controls were selected at random, matched on follow-up to the age of the case at diagnosis and, where appropriate, broad geographical region. Data for individual women were collated and analysed centrally so that analyses could be carried out using as similar definitions across studies as was possible. Details sought from principal investigators of each participating study included data that had been collected on each woman's reproductive history and various other factors that may be relevant to the aetiology of breast cancer, including the women's consumption of alcohol and tobacco.

Some investigators provided estimates of alcohol intake reported by each woman expressed as gram (g) of alcohol consumed per day or per week. Others provided information on the reported number of alcoholic drinks consumed daily or weekly. In such instances, the number of grams of alcohol consumed per day, was estimated assuming that one alcoholic drink contains 12 g alcohol in the USA and Italy,^11^ 8 g in the UK and 10 g elsewhere (Brewers' Society, personal communication). No information was sought about alcohol consumption at various ages or about the particular type of alcohol consumed. Information was also sought on whether or not each woman had ever smoked, and whether she was a current or past smoker. Active smoking only was considered and no attention given to the reported associations with environmental tobacco smoke,^35,49^ nor was information sought on the age women were when smoking started or stopped, or on the amount smoked. The methods of identifying studies and of data checking, and correction, have been described elsewhere.^64,65^

### Statistical analysis and presentation of results

Statistical methods were similar to those used in previous reports by this group.^64,65,66,67^ Data from different studies were combined by means of the Mantel–Haenszel stratification technique, the stratum-specific quantities calculated being the standard ‘observed minus expected’ (O–E) numbers of women with breast cancer, together with their variances and covariances. These values yield both statistical descriptions (odds ratios, subsequently referred to as relative risks) and statistical tests (*P* values). When only two groups are being compared, relative risk estimates are obtained from O–E values by the one-step method,^66^ as are their standard errors (SE) and confidence intervals (CI). When more than two groups are compared, variances are estimated by treating the relative risks as floating absolute risks (FARs).^67^ This approach yields floated standard errors (FSE) and floated confidence intervals (FCI). Presentation of the results in this way enables valid comparisons between any two exposure groups, even if neither is the baseline group. Any comparison between groups must take the variation in each estimate into account by summing the variances of the logarithms of the two FARs.

To obtain comparability between the women with breast cancer and similar women without breast cancer, all analyses were routinely stratified by study, and centre within study; by age (in single years from 16 to 64, 65 to 69, 70 to 74, etc., up to 85 to 89); by parity and, where appropriate, age when the first child was born (nulliparous women were assigned to a separate stratum and parous women were cross-classified according to parity (1–2, 3–4, 5–6, 7+) and age at first birth (<20, 20–24, 25–29, 30+)). Where appropriate analyses relating to alcohol consumption were stratified by smoking history (ever/never) and analyses relating to tobacco consumption were stratified by alcohol consumption (0, <5, 5–14, 15–24, 25–34, 35–44, ⩾45 g per day). In order to summarise the relationship between alcohol consumption and breast cancer risk, a linear trend in the log relative risk of breast cancer was fitted across increasing categories of consumption. In estimating such trends, the median consumption within a given category was taken to be the level of alcohol consumption for that category.

In general, results in the text are presented as relative risks and their appropriate SE or FSEs. Where results are presented in the form of plots, relative risks and their corresponding CIs/FCIs are represented by squares and lines, respectively. The position of the square indicates the value of the relative risk and its area is inversely proportional to the variance of the logarithm of the relative risk, thereby providing an indication of the amount of statistical information available for that particular estimate. Owing to the large number of relative risk estimates calculated, results are given with their appropriate 99% CIs/FCIs; and 95% CIs/FCIs are used only to summarise the main findings.

The absolute risk of breast cancer associated with various levels of alcohol consumption was estimated for women in developed countries, by applying the dose-response estimates obtained here to age-specific incidence rates for breast cancer in developed countries around 1990^64,65^ assuming that an intake of 10 g per day is roughly equivalent to one unit or drink of alcohol per day. The cumulative incidence of breast cancer up to age 80 years was calculated from the age-specific findings.

## RESULTS

The 65 studies that contributed individual data on alcohol and/or tobacco consumption and other factors relevant to breast cancer included a total of 66 426 women with invasive breast cancer (cases) and 126 953 women without breast cancer controls from 63 published^1,2,3,4,5,6,7,8,9,10,11,12,13,14,15,16,17,18,19,20,21,22,23,24,25,26,27,28,29,30,31,32,33,34,35,36,37,38,39,40,41,42,43,44,45,46,47,48,49,50,51,52,53,54,55,56,57,58,59,60,61,62,63^ and two unpublished studies. Information on both alcohol and tobacco had been collected in 53 of these studies, that included a total of 58 515 cases and 95 067 controls from 51 published^1,2,3,4,5,6,7,8,9,10,11,12,13,14,15,16,17,18,19,20,21,22,23,24,25,26,27,28,29,30,31,32,33,34,35,36,37,38,39,40,41,42,43,44,45,46,47,48,49,50,51^ and two unpublished studies. Unless otherwise specified, analyses presented here are restricted to data from these 53 studies. This enables women to be cross-classified by both their alcohol and tobacco consumption, thus permitting adequate examination of possible confounding between the two exposures.

Among women with breast cancer in the 53 studies included in the main analyses, the median year of diagnosis was 1988 and the average age at diagnosis was 52.1 years. All but five of the 53 studies^5,9,21,41,48^ were conducted in developed countries. Among controls, alcohol consumption was substantially greater in women from developed than developing countries (average alcohol intakes of 6.0 g per day and 0.4 g per day, respectively). The proportion of controls from developed countries who reported drinking no alcohol was 40%, and a further 28% reported consuming <5 g per day, i.e. less than half a unit/drink of alcohol per day (Table 1

**Table 1:**
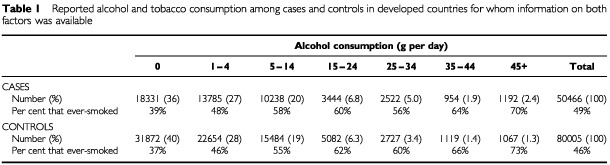
Reported alcohol and tobacco consumption among cases and controls in developed countries for whom information on both factors was available

). Only about 1% of the controls from developed countries reported drinking 35–44 g per day alcohol, i.e. about four units or drinks daily, and a similar proportion reported drinking ⩾45 g per day.

Overall about half the women in developed countries reported that they had ever smoked, but smoking habits varied considerably according to alcohol intake, both for cases and controls (Table 1). Among controls from developed countries who reported drinking no alcohol, 37% had ever smoked, and the proportion of ever-smokers increased with increasing intake of alcohol, rising to 73% for controls who reported drinking ⩾45 g per day alcohol (Table 1). The average alcohol consumption reported by ever-smokers from developed countries was greater than that reported by never-smokers (8.4 g per day *vs* 5.0 g per day).

Because alcohol and tobacco consumption are so closely associated, analyses of their effects were initially carried out separately for never-smokers and ever-smokers (in the case of alcohol) and for drinkers and non-drinkers (in the case of tobacco).

### Breast cancer in relation to alcohol consumption

Table 2

**Table 2:**
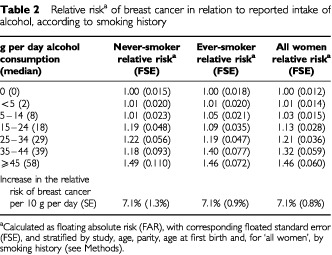
Relative risk^a^ of breast cancer in relation to reported intake of alcohol, according to smoking history

shows the relative risks and corresponding standard errors for breast cancer according to women's reported daily intake of alcohol for never-smokers and ever-smokers. In each group the relative risk of breast cancer increased significantly with increasing intake of alcohol, increasing by the same amount, 7.1%, for each additional 10 g per day intake of alcohol (*P*<0.00001 in each group). The trends associated with increasing levels of alcohol intake in never-smokers and ever-smokers did not differ significantly from each other (χ^2^_1_ for heterogeneity=0.002; *P*=1.0). Therefore subsequent analyses concerning alcohol consumption include both never-smokers and ever-smokers, and the data are stratified by smoking history as well as by study, age, parity and age at first birth.

**Figure 1:**
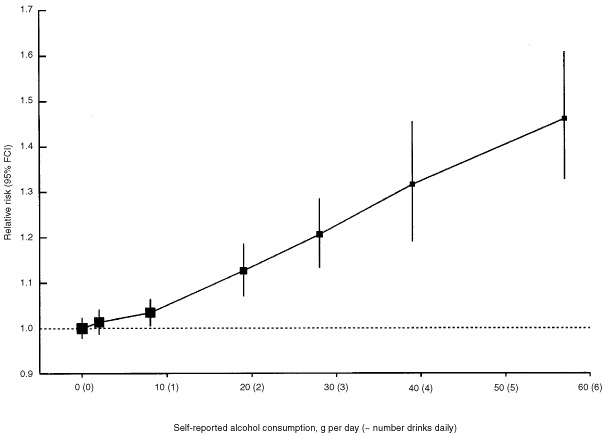
Relative risk of breast cancer in relation to reported intake of alcohol. Relative risks are calculated as floating absolute risk (FAR) and stratified by study, age, parity, age at first birth and smoking.

When the data in smokers and non-smokers were combined the relative risk of breast cancer increased with alcohol intake, increasing by 7.1% (SE 0.8%; *P*<0.00001) for each additional 10 g per day intake of alcohol, i.e. for each extra unit/drink of alcohol consumed on a daily basis (Figure 1

). Compared to women who drank no alcohol the relative risk was 1.32 (0.059, *P*<0.00001) for women whose reported alcohol consumption was 35–44 g per day and 1.46 (0.060, *P*<0.00001) for a consumption of ⩾45 g per day, where the average consumption was 57 g per day.

**Figure 2:**
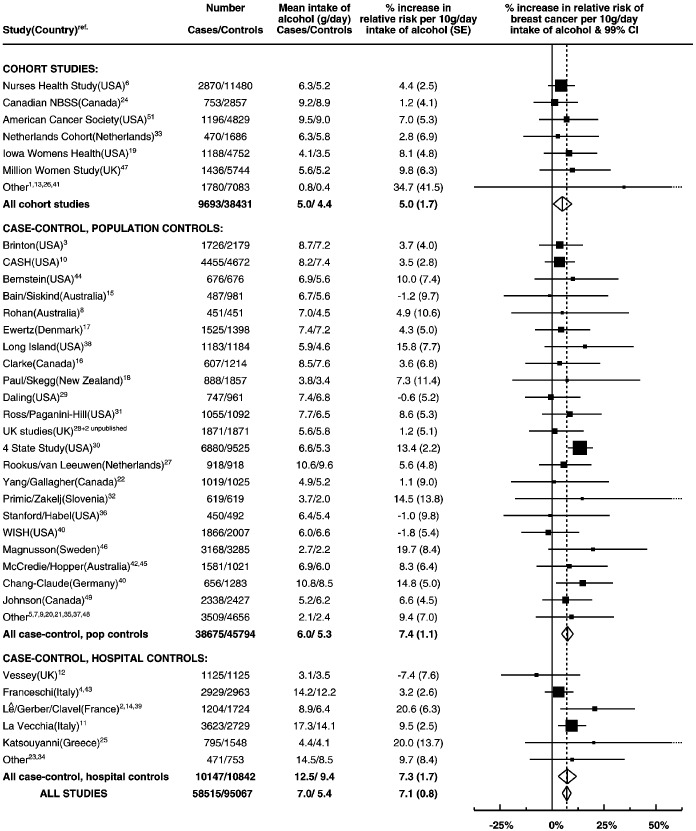
Details of and results from studies on the relation between alcohol consumption and breast cancer. Relative risks are stratified by age, parity, age at first birth and smoking history.

The study-specific results are summarised in Figure 2

, grouped according to study design. Studies which contributed the smallest amounts of statistical information, were grouped together as ‘other’ in each of these categories. There was no strong evidence to suggest that the results varied substantially across studies (χ^2^_52_=60.7; *P*=0.3) or according to study design (χ^2^_2_ for heterogeneity=1.5; *P*=0.5). In the one study^52^ which contributed data on alcohol, but not smoking, the estimated increase in the relative risk of breast cancer per additional 10 g per day intake was 13.8% (SE 10.5%). Because of the large standard error, the estimated increase in relative risk in this study does not differ significantly from results for all other studies combined (χ^2^_1_=0.4, *P*=0.5).

The effect of adjusting for 11 other potential confounding factors (race, education, family history of breast cancer, age at menarche, height, weight, body mass index, breastfeeding, use of hormonal preparations, and age at and type of menopause) on the relationship in Figure 1 is shown in Table 3

**Table 3:**
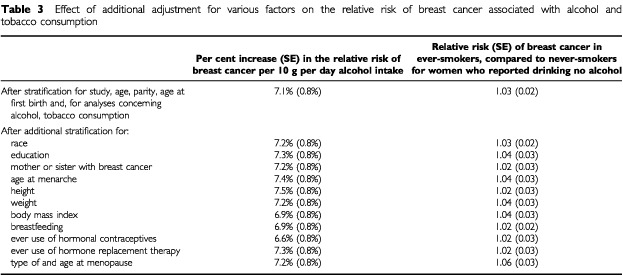
Effect of additional adjustment for various factors on the relative risk of breast cancer associated with alcohol and tobacco consumption

. Additional adjustment for each of these factors in turn did not materially alter the magnitude of the increase in the relative risk of breast cancer associated with increasing levels of alcohol intake, suggesting that the associations in Figure 1 are not much confounded by any of them.

### Breast cancer in relation to tobacco consumption

Among the 22 255 cases and 40 832 controls who reported drinking no alcohol, the risk of breast cancer in ever-smokers did not differ significantly from that in never-smokers (relative risk for ever *vs* never-smokers=1.03, SE 0.023; NS). However, among women who reported drinking alcohol, the findings for smoking were difficult to disentangle from the effects of the alcohol itself. When ever-smokers were compared to never-smokers the relative risk for breast cancer was 1.09 (0.018) before stratification by the amount of alcohol consumed, and declined to 1.05 (0.020) after stratification. The corresponding χ^2^_1_ value declined by three-quarters from 23.4 to 6.4. Since alcohol consumption is known to be unreliably measured,^68^ and stratification for such a poorly measured variable reduced the χ^2^ value by three-quarters, stratification by true alcohol intake would be expected to reduce the χ^2^ value by even more.^69^ Since it is not possible to eliminate residual confounding among drinkers, results concerning tobacco consumption are restricted to women who reported drinking no alcohol at all, where such confounding should be minimised.

**Figure 3:**
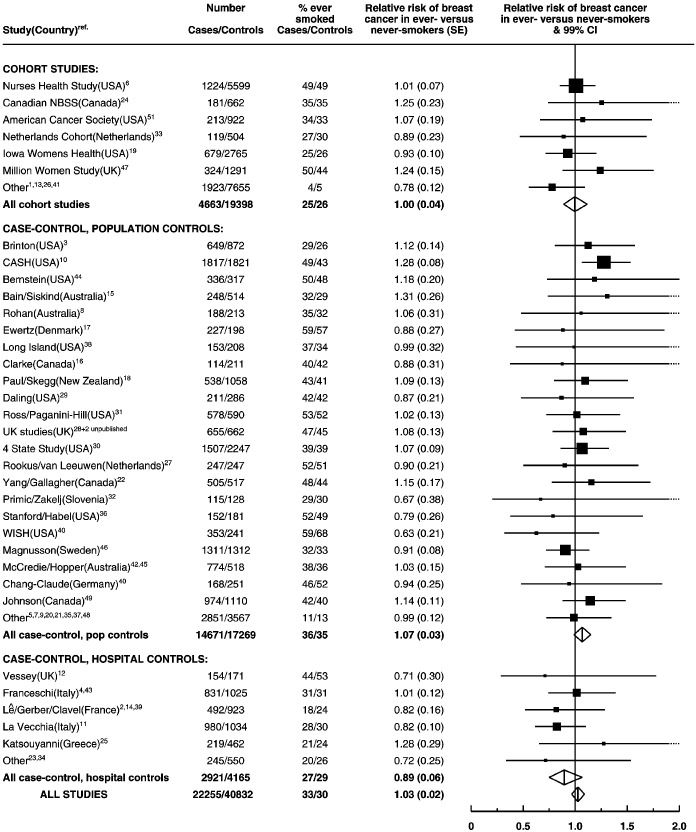
Details of and results on the relation between tobacco consumption and breast cancer in women who reported drinking no alcohol. Relative risks are stratified by age, parity and age at first birth.

The study-specific relative risks for breast cancer in ever-smokers compared to never-smokers are shown in Figure 3

, for women who reported drinking no alcohol. There was no marked variation in the relative risk of breast cancer across studies (χ^2^_52_=58.0, *P*=0.3) or study design (χ^2^_2_=6.1, *P*=0.05). Information on current and past smoking was available for all but five studies.^2,23,28^ (and two unpublished). Among ever-smokers in the remaining 48 studies 54% were current smokers and 46% were past smokers. Compared to never-smokers the relative risk of breast cancer was 0.99 (SE 0.03) for current smokers (Appendix III in Supplementary Material (www.bjcancer.com)), and 1.07 (SE 0.03) for past smokers (Appendix IV in Supplementary Material (www.bjcancer.com)).

Among controls from developed countries a greater proportion of ever-smokers than never-smokers had had a bilateral oophorectomy (8.7% *vs* 7.6%) or a hysterectomy without bilateral oophorectomy (13.3% *vs* 12.5%). The average age at bilateral oophorectomy was 41.6 (SD 7.5) and 44.2 (SD 6.6), respectively and the average age at hysterectomy was 38.6 (SD 9.3) and 40.0 (SD 9.9), respectively. Average age at natural menopause was also slightly earlier in ever-smokers than in never smokers, at 48.3 (SD 4.8) and 49.3 (SD 4.7) years, respectively. The relative risk of breast cancer in ever *vs* never-smokers was similar for women who had had an oophorectomy, hysterectomy or natural menopause (Table 4

**Table 4:**
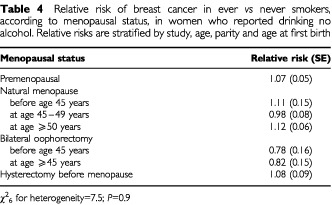
Relative risk of breast cancer in ever *vs* never smokers, according to menopausal status, in women who reported drinking no alcohol. Relative risks are stratified by study, age, parity and age at first birth

) and additional stratification by age at and type of menopause did not materially alter the overall magnitude of the relative risk (Table 3). Nor did additional stratification by 10 other potential confounding factors much alter the relative risk.

Eleven studies^53,54,55,56,57,58,59,60,61,62,63^ that together included a total of 4781 cases and 12 713 controls, contributed data to this collaboration on tobacco consumption for each woman, but not on alcohol consumption. The combined relative risk of breast cancer in ever-smokers compared to never-smokers in these 11 studies was 1.05 (SE 0.05), but because of the potential for confounding by alcohol the results from these studies have not been included in the main analyses.

### Consistency of the findings

**Figure 4:**
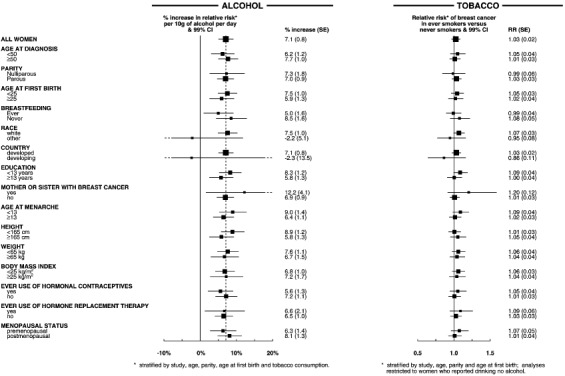
Relative risk of breast cancer in relation to alcohol and tobacco consumption in various subgroups of women.

The increase in the relative risk of breast cancer for each additional 10 g per day intake of alcohol consumption was calculated separately for various subgroups of women, subdivided according to 15 personal characteristics including their age, childbearing pattern, race and familial patterns of breast cancer. Overall there was no significant variation in the trend associated with increasing intake of alcohol between categories defined by any of the 15 factors examined (Figure 4

: global test for heterogeneity χ^2^_15_=18.0; *P*=0.3). Nor was there significant variation in the relative risk of breast cancer associated with having ever smoked across categories of the 15 characteristics examined (Figure 4: global test for heterogeneity χ^2^_15_=17.9; *P*=0.3).

Information on the extent of spread of the breast cancer was available for about 60% of the study population. Both for tumours localised to the breast and for tumours that had spread beyond the breast, the risk of breast cancer increased with increasing alcohol consumption (increase in relative risk of breast cancer of 6.9% (1.3%) and 9.4% (1.5%), respectively, per 10 g per day alcohol consumption: χ^2^_1_=3.3; *P*=0.07). There was no significant difference in the extent of tumour spread among the cases according to tobacco consumption (χ^2^_1_=3.0, *P*=0.08).

### Cumulative incidence of breast cancer

**Figure 5:**
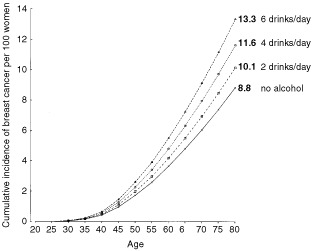
Estimated cumulative incidence of breast cancer per 100 women in developed countries, according to the number of alcoholic drinks consumed each day (see Methods).

Around 1990 the cumulative incidence of breast cancer up to age 80 years was between about eight and 10 per 100 women in developed countries.^64,65,70^ The average consumption of alcohol by controls studied here from developed countries was 6.0 g per day. If the dose-response relationship described here is valid, it is estimated that about 4% of breast cancers in developed countries are attributable to alcohol. The cumulative incidence of breast cancer by age 80 years is estimated to increase from 8.8 per 100 women in non-drinkers to 9.4, 10.1, 10.8, 11.6, 12.4 and 13.3, respectively, per 100 women consuming an average of 1, 2, 3, 4, 5 and 6 alcoholic drinks each day (see Figure 5

). In developing countries, where alcohol consumption is very low, averaging only about 0.4 g per day, alcohol would make a negligible contribution to the total number of cases of breast cancer.

## DISCUSSION

There is potential for confounding between the possible effects of alcohol and of tobacco on breast cancer, as drinking and smoking are closely associated, one with another. Among controls from developed countries, the proportion of ever-smokers rose from 37% in women who reported drinking no alcohol at all, to 73% in women drinking ⩾45 g per day alcohol, and alcohol consumption was greater in ever-smokers than in never-smokers, averaging 8.4 and 5.0 g per day, respectively.

The relative risk of breast cancer was found to increase with increasing intake of alcohol, both in never-smokers and in ever-smokers, and the magnitude of the increase was the same in each group (an increase of 7.1% in the relative risk of breast cancer for each additional 10 g per day alcohol; 95% CI 5.5–8.7% *P*<0.00001 overall). The observed association between breast cancer and alcohol consumption is therefore unlikely to be an indirect effect of tobacco.

Conversely, the relationship between smoking and breast cancer was found to be confounded by alcohol. Among women who drank no alcohol, ever-smokers and current smokers were not at an increased risk of breast cancer compared to never-smokers. Among women who drank alcohol, however, adjustment of the relative risk of breast cancer by the amount of alcohol consumed had a substantial effect on the results and, since it is not possible to measure alcohol intake reliably and thus eliminate residual confounding due to alcohol, we chose to base our assessment of the effect of tobacco on breast cancer on the 22 255 cases and 40 832 controls recorded as drinking no alcohol at all. In this large group of women the results suggest that smoking has little or no independent effect on the risk of developing breast cancer.

The association between breast cancer and alcohol or tobacco consumption does not appear to be materially confounded by the effects of other factors. Potential confounding by age, study, parity, age at first birth and tobacco consumption were minimised by stratification. Ever-smokers had their natural menopause about 1 year earlier, on average than never-smokers and were also more likely to have had a bilateral oophorectomy or hysterectomy, but adjustment for type of and age at menopause had little effect on the relative risk of breast cancer in ever- *vs* never-smokers (Tables 3 and 4). In addition, possible confounding by race, education, family history of breast cancer, age at menarche, height, weight, body mass index, breastfeeding and use of hormonal preparations was examined by adjustment for each factor in turn, but none materially altered the estimates of relative risk (Table 3). Since the relative risk estimates for breast cancer in relation to both alcohol and tobacco consumption did not appear to differ substantially according to any of these factors, there is no strong evidence for interaction between either of these exposures and the 15 factors examined (Figure 4).

There was no significant difference in the extent of tumour spread according to either alcohol or tobacco consumption, suggesting that there is little differential detection of breast cancer or effect on tumour growth by these exposures.

### Combining results from different studies

Combining results across many studies has the advantage of yielding estimates of the relative risk that are not subject to as much random fluctuation as that found in any individual study. The studies that contributed to these findings were of different designs and included women with a wide range of alcohol and tobacco consumption and of other personal characteristics. Nevertheless, the relationships between breast cancer and alcohol and tobacco were seen consistently across studies and study designs, and for women of different ages, different childbearing histories, and for women who differed according to various other personal characteristics. The results were not unduly influenced by any particular study or groups of studies.

Because of the strong association between alcohol and tobacco consumption, the main analyses were restricted to data from the 53 studies in which information on both exposures had been collected in the same women. Results from the only study^52^ that had provided individual data on alcohol, but not tobacco, did not differ significantly from the overall findings for breast cancer and alcohol. The remaining 11 studies^53,54,55,56,57,58,59,60,61,62,63^ that provided individual data on tobacco, but not on alcohol, could not contribute directly to this review, since it was not possible to take into account for the important confounding effect of alcohol. None of the publications from these 11 studies has, however, claimed that smoking affected the risk of breast cancer.

As far as can be ascertained, over 80% of the worldwide epidemiological data that have been assembled on the relationship between breast cancer and alcohol and tobacco consumption were contributed to this collaboration. Another 20 studies were identified with relevant data that together included about 12 000 women with breast cancer (see Appendix II in Supplementary Material (www.bjcancer.com)), but because results were presented in a different way in each study, it is difficult to combine the published data directly. Nevertheless, out of the six largest studies all but one (reference number 66, in Appendix II Supplementary Material (www.bjcancer.com)) reported a statistically significant increased risk of breast cancer with increasing intake of alcohol. Each of these six studies included at least 500 women with breast cancer and altogether they comprised most of the information that had not been contributed in this collaboration. The remaining 14 studies were comparatively small and none of their published results on alcohol differed substantially from those reported here. Therefore the findings on alcohol and breast cancer from studies not included here do not appear to differ materially from these results.

Only one of the 20 studies that had not contributed to this collaboration claimed that smoking is associated with an increased risk breast cancer (reference number 81, in Appendix II Supplementary Material (www.bjcancer.com)). None of these studies has, however, published results on the risk of breast cancer in relation to smoking, restricted to women who never drank alcohol.

### Limitations of these findings

Overall, the relative risk of breast cancer appeared to increase by 7.1% (95% CI 5.5–8.7%) for each additional 10 g per day intake of alcohol i.e. for each extra unit/drink of alcohol consumed on a daily basis. Information on alcohol consumption was, however, usually self-reported, describing drinking habits at around the time that the women were interviewed. No information on the pattern of intake, including the type of alcohol consumed and the duration of intake, was collected for this collaboration. There is no strong evidence here to suggest biased reporting of alcohol consumption in case-control studies, since there was no significant difference in results between case–control and cohort studies (increases of 7.4% and 5.0% per 10 g per day, respectively; χ^2^_1_ for heterogeneity=1.5, *P*=0.2). However, self-reported information on alcohol consumption is known to underestimate true consumption.^68^ Systematic under-reporting of consumption by both cases and controls would result in an overestimation of the relative risk of breast cancer for a given level of alcohol consumption. By contrast, random misclassification among both cases and controls would have the opposite effect, resulting in an underestimation of the relative risk. These two types of measurement error are inevitable, but counter-acting, and it is not possible to estimate their overall effect on the relative risks calculated here. Moreover, the shape of the dose-response relationship could be changed if, for example, heavy drinkers were more likely to under-report intake than moderate drinkers. Taken together, these reporting errors imply that some uncertainty remains about the true quantitative effect of an intake of a fixed amount of alcohol on the risk of developing breast cancer.

The true relationship between alcohol consumption and breast cancer might, perhaps, be more curved than is suggested by the shape of the relationship shown in Figure 1, because of misclassification of alcohol intake, as may also have occurred with cigarette smoking and lung cancer.^71^ Any firm conclusion about the risk of breast cancer at low levels of alcohol intake is, however, prohibited by the likelihood of measurement errors, particularly the tendency for underestimation of the amount drunk, and by the possibility that non-drinkers may differ in some relevant, but unmeasured, ways from those who sometimes drink alcohol. Hence, the possibility of a threshold dose of alcohol cannot be reliably assessed from the data in Figure 1.

These results provide no direct evidence about possible mechanisms of carcinogenesis by alcohol on the breast. There is, however, accumulating evidence that regular intakes of moderate amounts of alcohol affect sex hormone levels. For example, the results of a recently published small randomised trial of 51 postmenopausal women suggested that sex hormone levels may be increased after the consumption of 30 g per day alcohol for 8 weeks,^72^ levels of consumption that are associated here with a clear excess risk of breast cancer.

With respect to the consumption of tobacco, the main exposure variable examined here was whether or not a woman had ever smoked. No information was collected for this collaboration on the amount smoked or on the age that smoking started or stopped, nor has attention been given to the reported effects of environmental exposure to tobacco,^35,49^ as active smoking only has been considered. Although some past smokers may have smoked relatively infrequently, current smokers are likely to have had substantial lifetime exposures to tobacco, particularly in countries where lung cancer rates in women are high. Just over half the ever-smokers included in these analyses were current smokers, and among them the risk of breast cancer was similar to that in never-smokers (relative risk=0.99 (95% CI, 0.96–1.03)). The findings from case–control studies could, in theory, be biased if women with breast cancer stopped smoking when they first developed symptoms, or if there were differential reporting of smoking by cases and controls. However, the results from cohort studies, where exposure information was collected prospectively, suggest no increase in the risk of breast cancer in ever-smokers or current smokers compared to never-smokers (relative risk=1.00, 95% CI 0.93–1.07, for ever-smokers; and =0.94, 95% CI 0.84–1.05, for current smokers).

### Public health implications

If the pattern of breast cancer associated with increasing levels of alcohol consumption estimated here is valid, then about 4% of the breast cancers in women in developed countries may be attributable to alcohol. The consumption of alcohol by most women in developed countries is relatively low, with about two-thirds consuming little or no alcohol each day. For women in developed countries who regularly drink alcohol, the lifetime risk of breast cancer is estimated to increase by about 0.7 per 100 women for each extra unit or drink of alcohol consumed on a daily basis. For example, the cumulative incidence of breast cancer by age 80 years is estimated to increase from 8.8 per 100 women who drink no alcohol to 10.1 or 100 who consume two alcoholic drinks daily and to 11.6 per 100 who consume four drinks daily. This excess risk should be considered in the context of the beneficial effects of alcohol, in moderation, on cardiovascular disease, and its harmful effects on cirrhosis and on cancers of the mouth, larynx, oesophagus and the liver.^73,74^

## APPENDIX I

### COLLABORATORS (in alphabetical order of institution, study name, or location)

*Aichi Cancer Research Institute, Nagoya, Japan:* **N Hamajima**, **K Hirose**, **K Tajima;** *Albert Einstein College of Medicine, NY, USA:* **T Rohan**; *American Cancer Society, GA, USA*: **EE Calle**, **CW Jr Heath**; *Atlanta, Emory University, GA, USA*: **RJ Coates**, **JM Liff**; *Aviano Cancer Center, Pordenone, Italy*: **R Talamini**; *Mahidol University, Bangkok, Thailand*: **N Chantarakul**, **S Koetsawang**, **D Rachawat**; *Breast Tumor Collaborative Study, Johns Hopkins University, MD, USA*: **A Morabia**, **L Schuman**, **W Stewart**, **M Szklo**; *University of Queensland, Brisbane, Australia*: **C Bain**, **F Schofield**, **V Siskind**; *British Columbia Cancer Agency, BC, Canada*: **P Band**, **AJ Coldman**, **RP Gallagher**, **TG Hislop**, **P Yang**; *Cancer Research Center, University of Hawaii, Hawaii, USA*: **LM Kolonel**, **AMY Nomura**; *Canadian Cancer Registries Epidemiology Research Group, Canada*: **J Hu**, **KC Johnson**, **Y Mao**; *Catal*á*n Institut of Oncology, Barcelona, Spain*: **S De Sanjos**é; *Centers for Disease Control* &* Prevention, GA, USA*: **N Lee**, **P Marchbanks**, **HW Ory**, **HB Peterson**, **HG Wilson**, **PA Wingo**; *Central Institute of Cancer Research, Berlin, Germany*: **K Ebeling**, **D Kunde**, **P Nishan**; *Centre for Genetic Epidemiology, University of Melbourne, Melbourne, Australia*: **JL Hopper**; *Channing Laboratory, Brigham and Women*'*s Hospital, Harvard Medical School, MA, USA*: **G Colditz for Nurses**' **Health Study Research Group**; *Chennai Cancer Institute, Madras, India*: **V Gajalakshmi**; *Chiang Mai University, Chiang Mai, Thailand*: **N Martin**, **T Pardthaisong**, **S Silpisornkosol**, **C Theetranont**; *Chulalongkorn University, Bangkok, Thailand*: **B Boosiri**, **S Chutivongse**, **P Jimakorn**, **P Virutamasen**, **C Wongsrichanalai**; *Danish Cancer Society, Aalborg, Denmark*: **M Ewertz**; *Department of Medical Epidemiology, Karolinska Institute, Stockholm, Sweden***:** **HO Adami**, **L Bergkvist**, **C Magnusson**, **I Persson**; *Deutsches Krebsforschungszentrum, Heidelberg, Germany*: **J Chang-Claude**; *University of Otago, Dunedin, New Zealand*: **C Paul**, **DCG Skegg**, **GFS Spears**; *European Institute of Oncology, Milan, Italy*: **P Boyle**, **T Evstifeeva**; *Fred Hutchinson Cancer Research Center, WA, USA*: **JR Daling**, **WB Hutchinson**, **K Malone**, **EA Noonan**, **JL Stanford**, **DB Thomas**, **NS Weiss**, **E White**; *French Multicentre Breast Study, INSERM, Villejuif, France*: **N Andrieu**, **A Br**ê**mond**, **F Clavel**, **B Gairard**, **J Lansac**, **L Piana**, **R Renaud**; *Girona Cancer Registry, Girona, Spain*: **A Izquierdo**, **P Viladiu**; *Hospital General de Mexico, Mexico City, Mexico*: **HR Cuevas**, **P Ontiveros**, **A Palet**, **SB Salazar**; *Hospital Universitario, Cali, Colombia*: **N Aristizabal**, **A Cuadros**; *Icelandic Cancer Society, Reykjavik, Iceland*: **L Tryggvadottir**, **H Tulinius**; *INSERM, Institut Gustave-Roussey, Villejuif, France*: **A Bachelot**, **MG L**ê; *Institute of Cancer Research, Sutton and London School of Hygiene and Tropical Medicine, UK*: **J Peto**; *International Agency for Research in Cancer, Lyon, France*: **S Franceschi**; *Israel Chaim Sheba Medical Centre, Tel-Hashomer, Israel*: **F Lubin**, **B Modan**, **E Ron**, **Y Wax**; *Kaiser Permanente, CA, USA*: **GD Friedman**, **RA Hiatt**; *Institut universitaire de medecine sociale et preventive, Lausanne, Switzerland***:** **F Levi**; *Cancer Research UK Genetic Epidemiology Laboratory, Leeds, UK*: **T Bishop**; *Institute of Oncology, Ljubljana, Slovenia*: **K Kosmelj**, **M Primic-Zakelj**, **B Ravnihar**, **J Stare**; *Loma Linda University, CA, USA*: **WL Beeson**, **G Fraser**; *Cancer Research UK Department of Mathematics, Statistics* &* Epidemiology, London*: **RD Bulbrook**, **J Cuzick**, **SW Duffy**, **IS Fentiman**, **JL Hayward**, **DY Wang**; *London School of Hygiene* & *Tropical Medicine, London, UK*: **AJ McMichael**, **K McPherson**; *Long Island Breast Cancer Study, NY, USA*: **RL Hanson**, **MC Leske**, **MC Mahoney**, **PC Nasca**, **AO Varma**, **AL Weinstein**; *University Hospital, Lund, Sweden*: **TR Moller**, **H Olsson**, **J Ranstam**; *Maastricht University, Maastricht, The Netherlands*: **RA Goldbohm**, **PA van den Brandt**; *University of Philippines, Manila, Philippines*: **RA Apelo**, **J Baens**, **JR de la Cruz**, **B Javier**, **LB Lacaya**, **CA Ngelangel**; *Istituto* ‘*Mario Negri*’*, Milan, Italy*: **C La Vecchia**, **E Negri**; *Istituto Nazionale Tumori, Divisione di Statistica Medica e Biometria, Milan, Italy***: E Marubini**; *Istituto di Statistica Medica e Biometria, Milan, Italy*: **M Ferraroni**; *Montpellier Cancer Centre* &* INSERM, Montpellier, France*: **M Gerber**, **S Richardson**, **C Segala**; *Nairobi Centre for Research in Reproduction, Nairobi, Kenya*: **D Gatei**, **P Kenya**, **A Kungu**, **JG Mati**; *National Cancer Institute, MD, USA*: **LA Brinton**, **R Hoover**, **C Schairer**; *National Institute of Child Health* &* Human Development, MD, USA*: **R Spirtas**; *National University of Singapore, Singapore*: **HP Lee**; *The Netherlands Cancer Institute, Amsterdam, The Netherlands***:** **MA Rookus**, **FE van Leeuwen for the Netherlands Oral Contraceptives and Breast Cancer Study Group**; *New Jersey State Department of Health, NJ, USA*: **JA Schoenberg**; *New South Wales Cancer Council, Sydney, Australia*: **M McCredie**; *Columbia University School of Public Health, NY, USA*: **MD Gammon**; *Ontario Cancer Treatment* &* Research Foundation, Ontario, Canada*: **EA Clarke**; *Department of Public Health* &* Primary Care, Oxford, UK*: **L Jones**, **A Neil**, **M Vessey**, **D Yeates**; *Cancer Research UK Epidemiology Unit, Oxford, UK (Secretariat)***:** **P Appleby**, **E Banks**, **V Beral**, **D Bull**, **B Crossley**, **A Goodill**, **J Green**, **C Hermon**, **T Key**, **N Langston**, **C Lewis**, **G Reeves**; *Cancer Research UK/MRC/BHF Clinical Trial Service Unit* &* Epidemiological Studies Unit, Oxford, UK*: **R Collins**, **R Doll**, **R Peto**; *Radiation Effects Research Foundation, Hiroshima, Japan*: **K Mabuchi**, **D Preston**; *Royal College of General Practitioners Oral Contraception Study, London, UK*: **P Hannaford**, **C Kay**; *University of Costa Rica, San Jose, Costa Rica*: **L Rosero-Bixby**; *Shanghai Cancer Institute, Shanghai, China*: **YT Gao**, **F Jin**, **J-M Yuan**; *Shanghai Institute of Planned Parenthood Research, Shanghai, China*: **HY Wei**, **T Yun**, **C Zhiheng**; *Department of Public Health, Sydney, Australia*: **G Berry**, **J Cooper Booth**, **T Jelihovsky**, **R MacLennan**, **R Shearman**; *Tianjin Cancer Institute, Tianjin, China*: **Q-S Wang**; *Department of Public Health Sciences, Toronto, Canada*: **CJ Baines**, **AB Miller**, **C Wall**; *Tromso University, Tromso, Norway*: **E Lund**, **H Stalsberg**; *Vanderbilt University, TN, USA:* **XO Shu**, **W Zheng**; *University of Athens Medical School, Athens, Greece*: **K Katsouyanni**, **A Trichopoulou**, **D Trichopoulos**; *University of Chile, Santiago, Chile*: **A Dabancens**, **L Martinez**, **R Molina**, **O Salas**; *University of Edinburgh, Edinburgh, UK*: **FE Alexander**; *University of Minnesota School of Public Health, MN, USA*: **K Anderson**, **AR Folsom on behalf of the Iowa Women**'**s Health Study**; *University of North Carolina at Chapel Hill, School of Public Health, NC, USA*: **BS Hulka**; *University of Nottingham, Nottingham, UK*: *CED Chilvers; University of Southern California, LA, USA*: **L Bernstein**, **S Enger**, **RW Haile**, **A Paganini-Hill**, **MC Pike**, **RK Ross**, **G Ursin**, **MC Yu**; *University of Wisconsin Comprehensive Cancer Center, WI, USA*: **MP Longnecker**, **P Newcomb for the 4 State Study**; *Vasteras, Sweden*: **L Bergkvist**; *World Health Organisation, Geneva, Switzerland*: **A Kalache**; *World Health Organisation, UNDP/UNFPA/WHO/World Bank Special Programme of Research, Development and Research Training in Human Reproduction, Geneva, Switzerland*: **TMM Farley**, **S Holck**, **O Meirik**.

Analysis and writing committee: Beral V, Bull D, Doll R, Peto R, Reeves G

Steering committee: Skegg D (Chairman), Colditz G, Hulka B, La Vecchia C, Magnusson C, Muller A, Peterson B, Pike M, Thomas D, Van Leeuven M.

## APPENDIX II

### References to epidemiological studies of breast cancer and alcohol and tobacco consumption and to reviews of the topic

See Supplementary Information

## APPENDIX III

### Results on the relation between current smoking and breast cancer in women who reported drinking no alcohol

See Supplementary Information

## APPENDIX IV

### Results on the relation between past smoking and breast cancer in women who reported drinking no alcohol

See Supplementary Information

## Supplementary Information

Appendix II References to epidemiological studies of breast cancer and alcohol and tobacco consumption and to reviews of the topic.

Appendix III Results on the relation between current smoking and breast cancer in women who reported drinking no alcohol. Relative risks are stratified by age, parity and age at first birth.

Appendix IV Results on the relation between past smoking and breast cancer in women who reported drinking no alcohol. Relative risks are stratified by age, parity and age at first birth.
