# Supplementary material for: Alcohol, tobacco and breast cancer – collaborative reanalysis of individual data from 53 epidemiological studies, including 58 515 women with breast cancer and 95 067 women without the disease
Source: Br J Cancer. 2002 Nov 12;87(11):1234–45. doi: 10.1038/sj.bjc.6600596 (PMC2562507; doi:10.1038/sj.bjc.6600596)
Supplement: Appendix II — References to epidemiological studies of breast cancer and alcohol and tobacco consumption and to reviews of the topic. [file 87-6600596x1.pdf]

APPENDIX II. – References to epidemiological studies of breast cancer and alcohol and tobacco consumption and to reviews of the topic

2.1 Studies contributing to the Collaborative Group on Hormonal Factors in Breast Cancer, which collected information on both alcohol and tobacco for each woman

1. Hiatt RA, Bawol RD. (1984). Alcoholic beverage consumption and breast cancer incidence. *Am J Epidemiol* **120:** 676–683
2. Lê MG, Moulton LH, Hill C, Kramar A. (1986). Consumption of dairy produce and alcohol in a case-control study of breast cancer. *J Natl Cancer Inst* **77:** 633–636
3. Harvey EB, Schairer C, Brinton LA, *et al.* (1987). Alcohol consumption and breast cancer. *J Natl Cancer Inst* **78:** 657–661
4. La Vecchia C, Decarli A, Parazzini F, Gentil A, Negri E, Cecchetti G, Franceschi S. (1987). General epidemiology of breast cancer in Northern Italy. *Int J Epidemiol* **16:** 347–355
5. Lee NC, Rosero-Bixby L, Oberle MW, Grimaldo C, Whatley AS, Rovira EZ. (1987). A case-control study of breast cancer and hormonal contraception in Costa Rica. *J Natl Cancer Inst* **79:** 1247–1254
6. Willett WC, Stampfer MJ, Colditz GA, *et al.* (1987). Moderate alcohol consumption and the risk of breast cancer. *N Engl J Med* **316:** 1174–1180
7. Adami HO, Lund E, Bergstrom R, *et al.* (1988). Cigarette smoking, alcohol consumption and risk of breast cancer in young women. *Br J Cancer* **58:** 832–837
8. Rohan TE & McMichael AJ. (1988). Alcohol consumption and risk of breast cancer. *Int J Cancer* **41:** 695–699
9. Yuan J-M, Yu MC, Ross RK, Gao Y-T & Henderson BE. (1988). Risk factors for breast cancer in Chinese women in Shanghai. *Cancer Res* **48:** 1949–1953
10. Chu SY, Lee NC, Wingo PA, Webster LA. (1989). Alcohol consumption and the risk of breast cancer. *Am J Epidemiol* **130:** 867–877
11. La Vecchia C, Negri E, Parazzini F, Boyle P, Fasoli M, Gentile A, Franceschi S. (1989). Alcohol and breast cancer: update from an Italian case-control study. *Eur J Cancer Clin Onco* **25 (12):** 1711–1717
12. Meara J, McPherson K, Roberts M, *et al.* (1989). Alcohol, cigarette smoking and breast cancer. *Br J Cancer* **60:** 70–73
13. Mills PK, Beeson WL, Phillips RL, Fraser GE. (1989). Prospective study of exogenous hormone use and breast cancer in Seventh-day Adventists. *Cancer* **64:** 591–597
14. Richardson S, De Vincenzi I, Pujol H, *et al.* (1989). Alcohol consumption in a case-control study of breast cancer in southern France. *Int J Cancer* **44:** 84–89
15. Siskind V, Schofield F, Rice D & Bain C. (1989). Breast cancer and breastfeeding: results from an Australian case-control study. *Am J Epidemiol* **130:** 229–236
16. Rosenberg L, Palmer JR, Miller DR, *et al.* (1990). A case-control study of alcoholic beverage consumption and breast cancer. *Am J Epidemiol* **131:** 6–14
17. Ewertz M. (1991). Alcohol consumption and breast cancer risk in Denmark. *Cancer Causes and Control* **2:** *247–252*
18. Sneyd MJ, Paul C, Spears GF, Skegg DCG. (1991). Alcohol consumption and risk of breast cancer. *Int J Cancer* **48:** 812–815
19. Gapstur SM, Potter JD, Sellers TA, Folsom AR. (1992). Increased risk of breast cancer with alcohol consumption in postmenopausal women. *Am J Epidemiol* **136:** 1221–1231
20. Ursin G, Aragaki CC, Paganini-Hill A, Siemiatycki J, Thompson WD, Haile RW. (1992). Oral contraceptives and premenopausal bilateral breast cancer: a case-control study. *Epidemiology* **2:** 414–419
21. Wang Q-S, Ross RK, Yu MC, Ning J-P, Henderson BE & Kimm HT. (1992). A case-control study of breast cancer in Tianjin, China. Cancer Epidemiology, *Biomarkers and Prevention* **1:** 435–439
22. Yang CP, Daling JR, Band PR, Gallagher RP, White E & Weiss NS. (1992). Non contraceptive hormone use and risk of breast cancer. *Cancer, Causes and Control* **3:** 475–479
23. Ferraroni M, Gerber M, Decarli A, *et al.* (1993). HDL-cholesterol and breast cancer: a joint study in Northern Italy and Southern France. *International Journal of Epidemiology* **22:** 772–780
24. Friedenreich CM, Howe GR, Miller AB. (1993). A cohort study of alcohol consumption and risk of breast cancer. *Am J Epidemiol* **137:** 512–520
25. Katsouyanni K, Trichopoulou A, Stuver S, Vassilaros S, Papadiamantis Y, Bournas N, Skarpou N, Mueller N, Trichopoulos D. (1994). Ethanol and breast cancer: an association that may be both confounded and causal. *Int J Cancer* **58:** 356–361
26. Land CE, Hayakawa N, Machado SG *et al.* (1994). A case-control interview study of breast cancer among Japanese A-bomb survivors. II. Interactions with radiation dose. *Cancer Causes and Control* **5:** 167–176
27. Rookus MA, van Leeuwen FE for the Netherlands Oral Contraceptives and Breast Cancer Study Group. (1994). Oral contraceptives and risk of breast cancer in women aged 20-54 years. *Lancet* **344:** 844–851
28. Smith SJ, Deacon JM, Chilvers CE. (1994). Alcohol, smoking, passive smoking and caffeine in relation to breast cancer risk in young women. UK National Case-Control Study Group. *Br J Cancer* **70:** 112–119
29. White E, Malone KE, Weiss NS, Daling JR. (1994). Breast cancer among young US women in relation to oral contraceptive use. *JNCI* **86 (7):** 505–514
30. Longnecker MP, Newcomb PA, Mittendorf R, Greenberg ER, Clapp RW, Bogdan GF, Baron J, MacMahon B, Willett WC. (1995). Risk of breast cancer in relation to lifetime alcohol consumption. *J Natl Cancer Inst* **87:** 923–929
31. Longnecker MP, Paganini-Hill A, Ross RK. (1995). Lifetime alcohol consumption and breast cancer risk among postmenopausal women in Los Angeles. *Cancer Epidemiology, Biomarkers & Prevention* **4:** 721–725
32. Primic-Zakelj M,Evstifeeva T, Ravnihar B, Boyle P. (1995). Breast cancer risk and oral contraceptive use in Slovenian women aged 25 to 54. *Int J Cancer* **62:** 414–420
33. van den Brandt PA, Goldbohm RA, van't Veer P. (1995). Alcohol and breast cancer: results from the Netherlands Cohort Study. *Am J Epidemiol* **141:** 907–915
34. Levi F, Pasche C, Lucchini F, La Vecchia C. (1996). Alcohol and breast cancer in the Swiss Canton of Vaud. *Eur J Cancer* **32A:** 2108–2113
35. Morabia A, Bernstein M, Heritier S Khatchatrian N (1996) Relation of breast cancer with passive and active exposure to tobacco smoke. *Amer J Epidemiol* **143:** 918–928
36. Rossing MA, Stanford JL, Weiss NS & Habel LA. (1996). Oral contraceptive use and risk of breast cancer in middle-aged women. *Am J Epidemiol* **144:** 161–164
37. Viladiu P, Izquierdo A, de Sanjosé S, Bosch FX. (1996). A breast case-control study in Girona, Spain. Endocrine, familial and lifestyle factors. *Eur J Cancer Prevention* **5:** 329–335
38. Bowlin SJ, Leske MC, Varma A, Nasca P, Weinstein A, Caplan L (1997) Breast cancer risk and alcohol consumption: results from a large case-control study. *Int J Epidemiol* **26:** 915–923
39. Clavel-Chapelon F, Dormoy N, Guibout C (1997) Wine, beer abd tobacco consumption and the risk of breast cancer: results from a French case-control study. *J Epidemiol Biostat* **2:** 95–104
40. Swanson CA, Coates RJ, Malone KE, *et al.* (1997). Alcohol consumption and breast cancer risk among women under age 45 years. *Epidemiology* **8:** 231–237
41. Thomas DB, Gao DL, Self SG, Allison CJ, Tao Y, Mahloch J, *et al.* (1997). Randomized trial of breast self-examination in Shanghai: methodology and preliminary results. *JNCI* **89:** 355–365
42. McCredie MRE, Dite GS, Giles GG, Hopper JL. (1998). Breast cancer in Australian women under the age 40. *Cancer Causes and Control* **9:** 189–198
43. Ferraroni M, Decarli A, Franceschi S, La Vecchia C. (1998). Alcohol consumption and risk of breast cancer: a multicentre Italian case-control study. *Eur J Cancer* **34:** 1403–1409
44. Enger SM, Ross RK, Paganini-Hill A, Longnecker MP, Bernstein L. (1999). Alcohol consumption and breast cancer oestrogen and progesterone receptor status. *Br J Cancer* **79:** 1308–1314
45. Hopper JL, Chenevix-Trench G, Jolley D, Dite GS, Jenkins MA, Venter DJ, McCredie MRE, Giles GG. (1999). Design and analysis issues in a population-based case-control-family study of the genetic epidemiology of breast cancer, and the Co-operative Family Registry for Breast Cancer Families (CFRBCS). *Monographs of the National Cancer Institute* **26:** 95–100
46. Magnusson C, Baron JA, Correia N, Bergstrom R, Adami H-O, Persson I. (1999). Breast cancer risk following long-term oestrogen- and oestrogen-progestin-replacement therapy. *Int J Cancer* **81:** 339–344
47. Million Women Study Collaborative Group. (1999). The Million Women Study: Design and Characteristics of the Study Population. *Breast Cancer Research* **1(1):** 73–80
48. Gao Y-T, Shu XO, Dai Q, Potter J, Brinton L, Wen W, Sellers T, Kushi LH, Yuen ZX, Bostick R, Jin F, Zheng W. (2000). Menstrual, reproductive factors and breast cancer risk in urban Shanghai, People's Republic of China. *Int J Cancer* **87:** 295–300
49. Johnson KC, Hu J, Mao Y & the Canadian Cancer Registries Epidemiology Research Group (2000) Passive and active smoking and breast cancer risk in Canada, 1994–97 *Cancer Causes and Control* **11:** 211–221
50. Kropp S, Becker H, Nieters A, Change-Claude J (2001) Low and Moderate alcohol consumption and breast cancer risk by age 50 among women in Germany. *Amer J Epidemiol* **154:** 624–34

© 2002 Cancer Research UK

51. Feigelson HS, Calle EE, Robertson AS, Wingo PA, Thun MJ (2001) Alcohol consumption increases the risk of fatal breast cancer. *Cancer Causes and Control* **12:** 895 – 902

2.2 Studies contributing to the Collaborative Group on Hormonal Factors in Breast Cancer, which collected information about alcohol but not tobacco

52. Thomas DB, Noonan EA and the WHO Collaborative Study of Neoplasia and Steroid Contraceptives. (1993). Breast cancer and prolonged lactation. *International Journal of Epidemiology* **22:** 619 – 626

2.3 Studies contributing to the Collaborative Group on Hormonal Factors in Breast Cancer, which collected information about tobacco but not alcohol

53. Vessey MP, McPherson K, Doll R. (1981). Breast cancer and oral contraceptives: findngs in Oxford-Family Planning Association contraceptive study. *Br Med J* **282:** 2093 – 2094
54. Pike MC, Henderson BE, Krailo MD, Duke A, Roy S. (1983). Breast cancer and oral contraceptives: reply to critics. *Lancet* December 17, 1414.
55. Nomura AMY, Hirohata T, Kolonel LN, Hankin JH, Lee J, Stemmermann G. (1985). Breast cancer in caucasian and Japanese women in Hawaii. *Natl Cancer Inst Monogr* **69:** 191 – 196
56. Hislop TG, Coldman AJ, Elwood JM, Skippen DH, Kan L. (1986). Relationship between risk factors for breast cancer and hormonal status. *Int J Epidemiol* **15:** 469 – 476
57. Kay CR, Hannaford PC. (1988). Breast cancer and the pill - a further report from the Royal College of General Practitioners' oral contraception study. *Br J Cancer* **58:** 675 – 680
58. Ravnihar B, Primic Zakelj M, Kosmelj K, Stare J. (1988). A case-control study of breast cancer in relation to oral contraceptive use in Slovenia. *Neoplasma* **35:** 109 – 121
59. Lee HP, Gourley L, Duffy SW, Esteve J, Lee J, Day NE. (1992). Risk factors for breast cancer by age and menopausal status: a case-control study in Singapore. *Cancer Causes & Control* **3:** 313 – 322
60. Ngelangel CA, Lacaya LB, Cordero C, Laudico AV. (1994). Risk factors for breast cancer among Filipino women. *Phil J Internal Med* **32:** 231 – 236
61. Hirose K, Tajima K, Hamajima N, Inoue M, Takezaki T, Kuroishi T, Yoshida M, Tokudome S. (1995). A large scale, hospital based case-control study of risk factors of breast cancer according to menopausal status. *Jpn J Cancer Res* **86 (146):** 154.
62. Thomas HV, Key TJ, Allen DS, Moore JW, Dowsett M, Fentiman IS, and Wang DY. (1997). Reversal of relation between body mass and endogenous estrogen concentrations with menopausal status. *JNCI* **89:** 396 – 397
63. Garrett PA, Hulka BS, Farber RA. (2000). Racial differences in the association of rare *HRAS* alleles and breast cancer. *Am J Epidemiol* **138 (8):** 599 – 599

2.4 Studies including ⩾500 women with breast cancer that did not contribute to the Collaborative Group on Hormonal Factors in Breast Cancer, (note: results both for alcohol and tobacco have been reported in these studies)

64. Rosenberg L, Slone D, Shapiro S, *et al.* (1982). Breast cancer and alcoholic-beverage consumption. *Lancet* **1:** 267 – 270
65. Garfinkel L, Boffetta P, Stellman SD. (1988). Alcohol and breast cancer: a cohort study. *Prev Med* **17:** 686 – 693
66. Harris RE, Wynder EL. (1988). Breast cancer and alcohol consumption: a study in weak associations. *JAMA* **259:** 2867 – 2871
67. Nasca P, Baptiste MS, Field NA, Metzger BB, Black M, Kwon CS, Jacobson H. (1990). An epidemiological case-control study of breast cancer and alcohol consumption. *International Journal of Epidemiology* **19(3):** 532 – 538
68. Martin-Moreno JM, Boyle P, Gorgojo L, *et al.* (1993). Alcoholic beverage consumption and risk of breast cancer in Spain. *Cancer Causes Control* **4:** 345 – 353
69. Freudenheim JL, Marshall JR, Graham S, Laughlin R, Vena JE, Swanson M, Ambrosone C, Nemoto T. (1995). Lifetime alcohol consumption and risk of breast cancer. *Nutrition and Cancer* **23:** 1 – 11

2.5 Studies including <500 women with breast cancer that did not contribute to the Collaborative Group on Hormonal Factors in Breast Cancer

70. Schatzkin A, Jones DY, Hoover RN, *et al.* (1987). Alcohol consumption and breast cancer in the epidemiologic follow-up study of the first National Health and Nutrition Examination Survey. *N Engl J Med* **316:** 1169 – 1173
71. Dupont WD, Page DL, Rogers LW, Parl FF. (1988). Influence of exogenous estrogens, proliferative breast disease, and other variables on breast cancer risk. *Cancer* **63:** 948 – 975
72. Iscovich JM, Iscovich RB, Howe G, Shiboski S, Kaldor JM. (1989). A case-control study of diet and breast cancer in Argentina. *Int J Cancer* **44:** 770 – 776
73. Toniolo P, Riboli E, Protta F, Charrel M, Cappa APM. (1989). Breast cancer and alcohol consumption: a case-control study in Northern Italy. *Cancer Research* **49:** 5203 – 5206
74. Young TB. (1989). A case-control study of breast cancer and alcohol consumption habits. *Cancer* **64:** 552 – 558
75. Zaridze D, Lifanova Y, Maximovitch D, *et al.* (1991). Diet, alcohol consumption and reproductive factors in a case-control study of breast cancer in Moscow. *Int J Cancer* **48:** 493 – 501
76. Graham S, Zielezny M, Marshall J, *et al.* (1992). Diet in the epidemiology of postmenopausal breast cancer in the New York State Cohort. *Am J Epidemiol* **136:** 1327 – 1337
77. Holmberg L, Baron JA, Byers T, Wolk A, Ohlander E-M, Zack M, Adami H-O. (1995). Alcohol intake and breast cancer risk: effect of exposure from 15 years of age. Cancer Epidemiology, *Biomarkers and Prevention* **4:** 843 – 847
78. Ranstam J, Olsson H. (1995). Alcohol, cigarette smoking, and the risk of breast cancer. *Cancer Detection and Prevention* **19(6):** 487 – 493
79. Royo-Bordonada MA, Martin-Moreno JM, Guallar E, *et al.* (1997). Alcohol intake and risk of breast cancer: The EURAMIC Study. *Neoplasma* **44:** 150 – 156
80. Viel J-F, Perarnau J-M, Challier B, Faivre-Nappez I. (1997). Alcoholic calories, red wine consumption and breast cancer among premenopausal women. *Eur J Epidemiol* **13:** 639 – 643
81. Lash TL, Aschengrau A (1999) Active and passive smoking and the occurrence of breast cancer. *Am J Epidemiol* **149:** 5 – 12
82. Zhang Y, Kreger BE, Dorgan JF, *et al.* (1999). Alcohol consumption and risk of breast cancer: the Framingham Study revisited. *Am J Epidemiol* **149:** 93 – 101
83. Manjer J, Berglund G, Bondesson L, Garne JP, Janzon L, Malina J. (2000). Breast cancer incidence in relation to smoking cessation. *Breast Cancer Research and Treatment* **61(2):** 121 – 129
84. Marcus PM, Newman P, Millikan RC, Moorman PG, Baird DD, Qaqish E. (2000), The associations of adolescent cigarette smoking, alcoholic beverage consumption, environmental tobacco smoke, and ionizing radiation with subsequent breast cancer risk (United States). *Cancer Causes and Control* **11:** 271 – 278

2.6 Selected reviews on breast cancer and alcohol/ tobacco consumption
Alcohol

85. IARC monographs on the evaluation of carcinogenic risks to humans. Vol. 44. Alcohol drinking. Lyon, France: International Agency for Research on Cancer, 1988.
86. Longnecker MP, Berlin JA, Orza MJ, Chalmers TC. (1988). A meta-analysis of alcohol consumption in relation to risk of breast cancer. *JAMA* **260:** 652 – 656
87. Smith-Warner SA, Spiegelman D, Yaun S-S, *et al.* (1998). Alcohol and breast cancer in women. *JAMA* **279:** 535 – 540

Tobacco

88. Baron JA. (1984). Smoking and estrogen-related disease. Am J Epidemiol **119:** 9 – 22
89. IARC monographs on the evaluation of carcinogenic risks of chemicals to humans. Vol. 38. Tobacco Smoking. Lyon, France: International Agency for Research on Cancer, 1986.
90. MacMahon B. (1990). Cigarette smoking and cancer of the breast. Smoking and hormone related disorders. In: Wald N, Baron J (editors). Oxford University Press, Oxford pp 154 – 166
91. Palmer JR, Rosenberg L. (1993). Cigarette smoking and the risk of breast cancer. *Epidemiologic Reviews* **15:** 145 – 156
92. Morabia A. (2002). Smoking (active and passive) and breast cancer: epidemiologic evidence up to June 2001. *Environ Mol Mutagen* **39:** 89 – 95

© 2002 Cancer Research UK
