# Supplementary figures and images for: Alcohol, tobacco and breast cancer – collaborative reanalysis of individual data from 53 epidemiological studies, including 58 515 women with breast cancer and 95 067 women without the disease
Source: Br J Cancer. 2002 Nov 12;87(11):1234–45. doi: 10.1038/sj.bjc.6600596 (PMC2562507; doi:10.1038/sj.bjc.6600596)

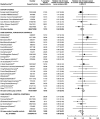

Supplement: Appendix III — Results on the relation between current smoking and breast cancer in women who reported drinking no alcohol. Relative risks are stratified by age, parity and age at first birth. [file 87-6600596x2.gif]

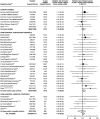

Supplement: Appendix IV — Results on the relation between past smoking and breast cancer in women who reported drinking no alcohol. Relative risks are stratified by age, parity and age at first birth. [file 87-6600596x3.gif]
